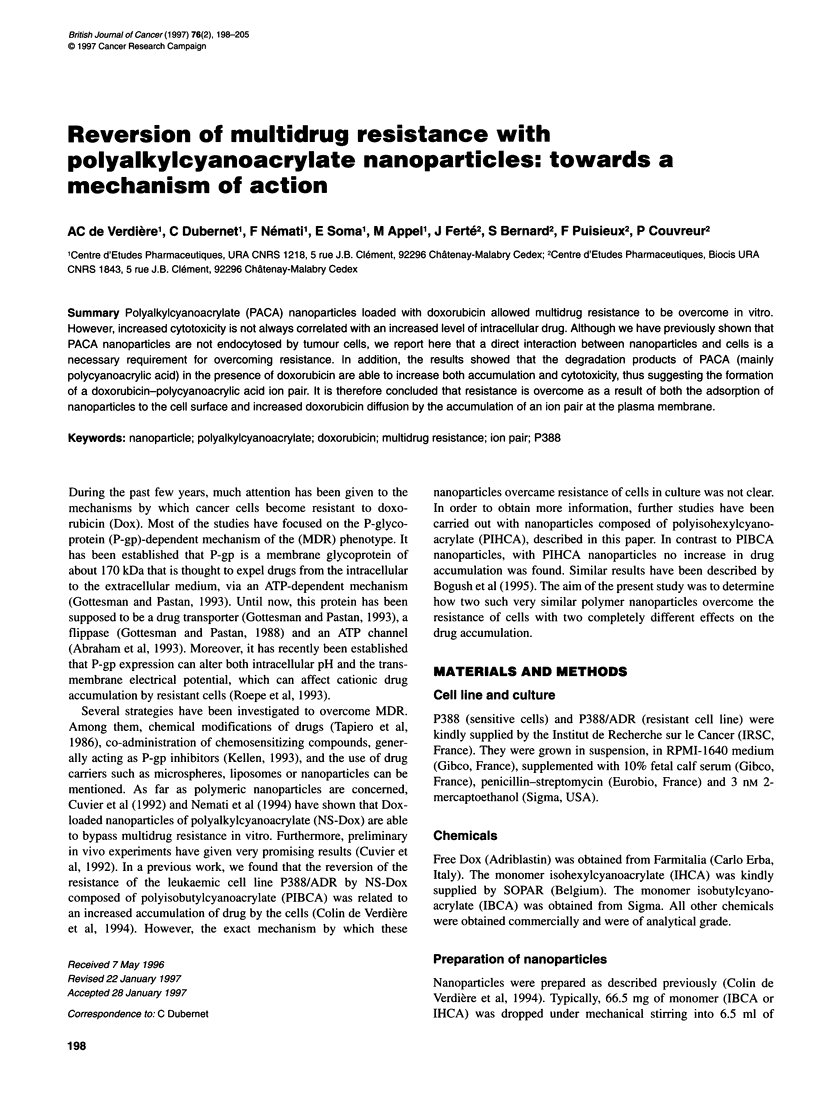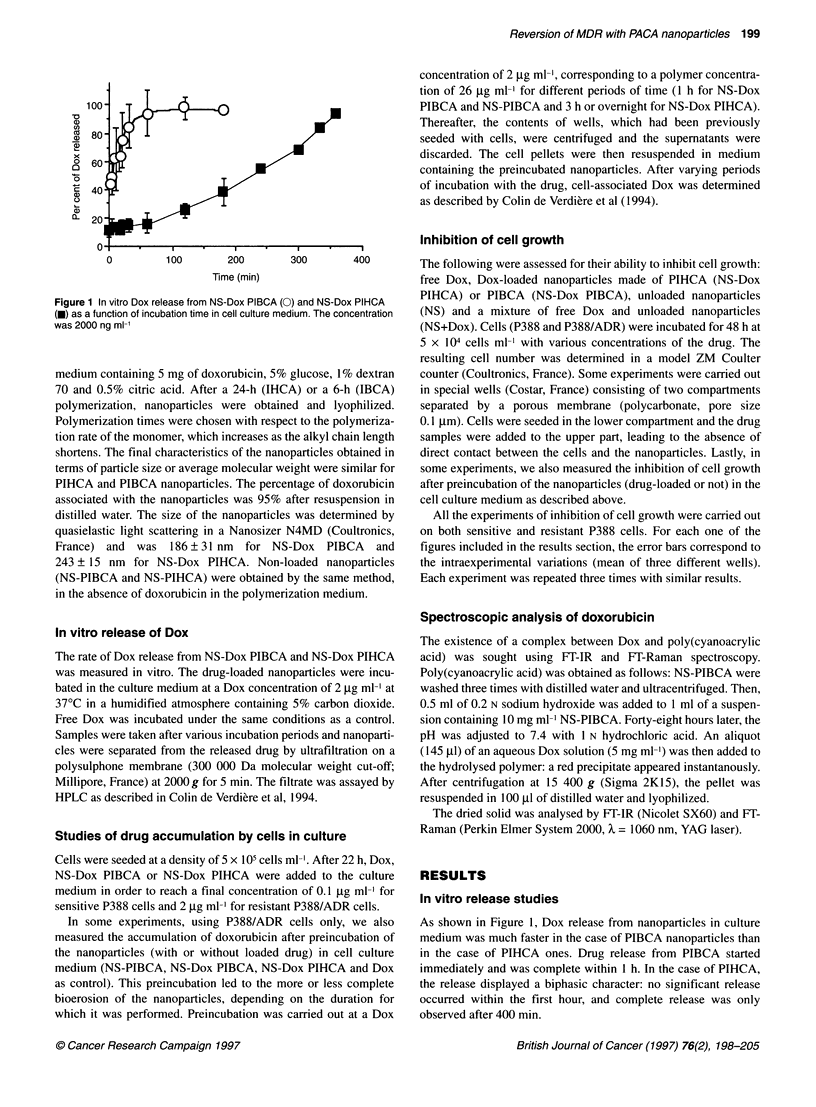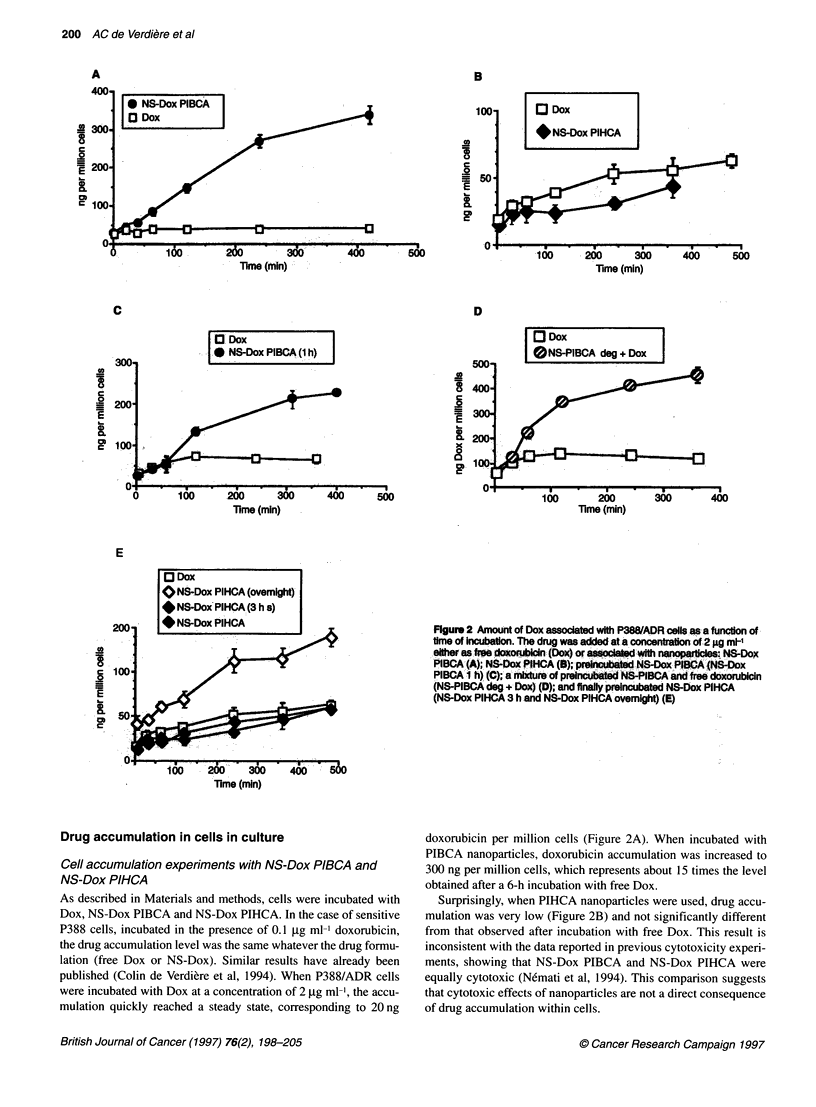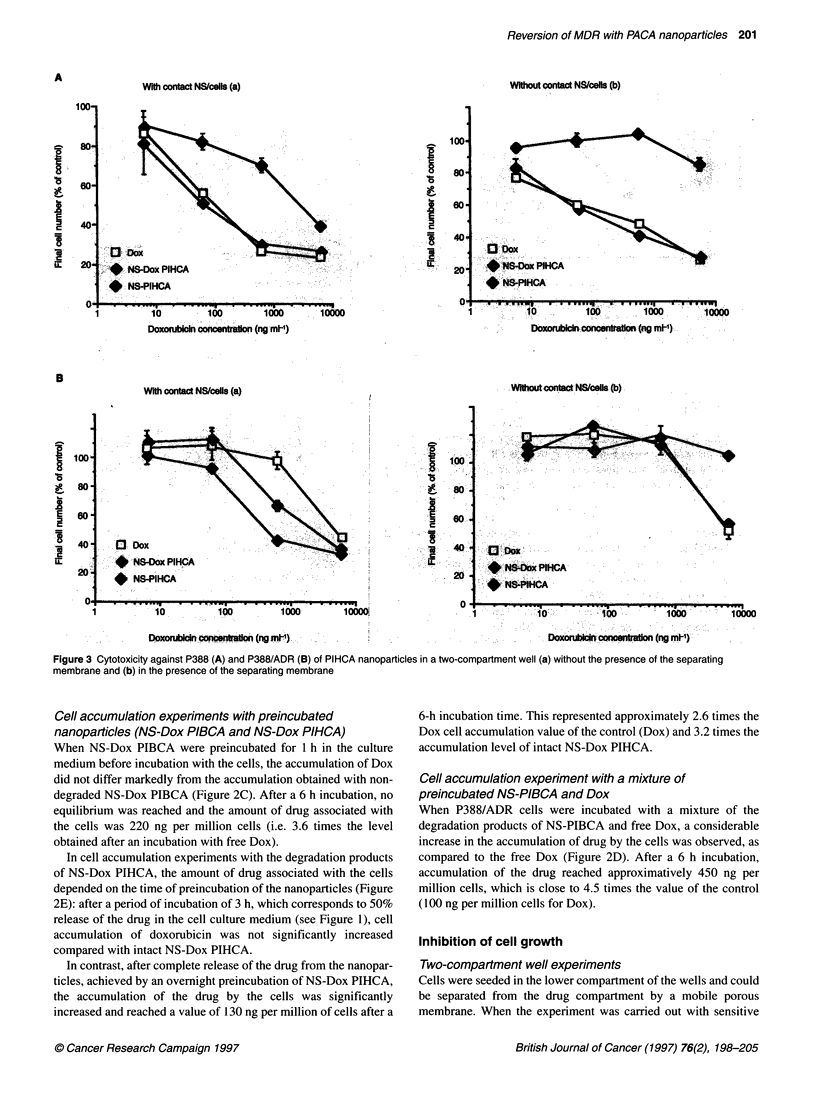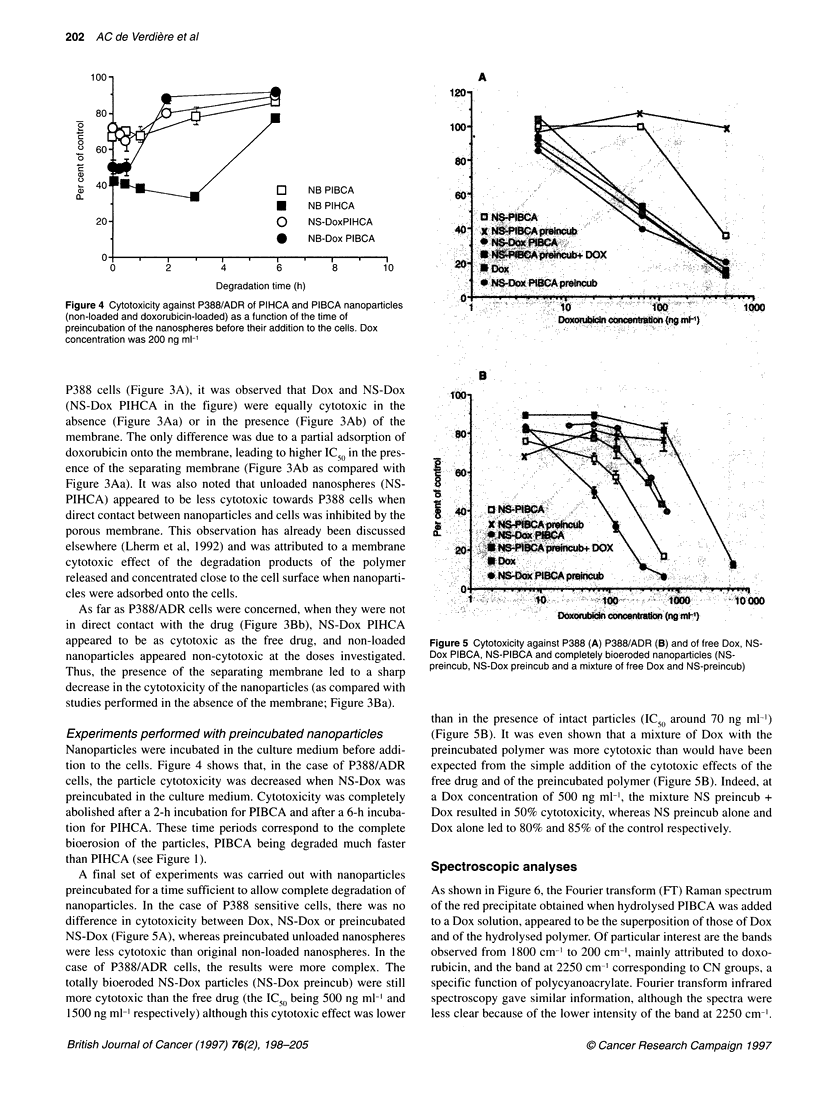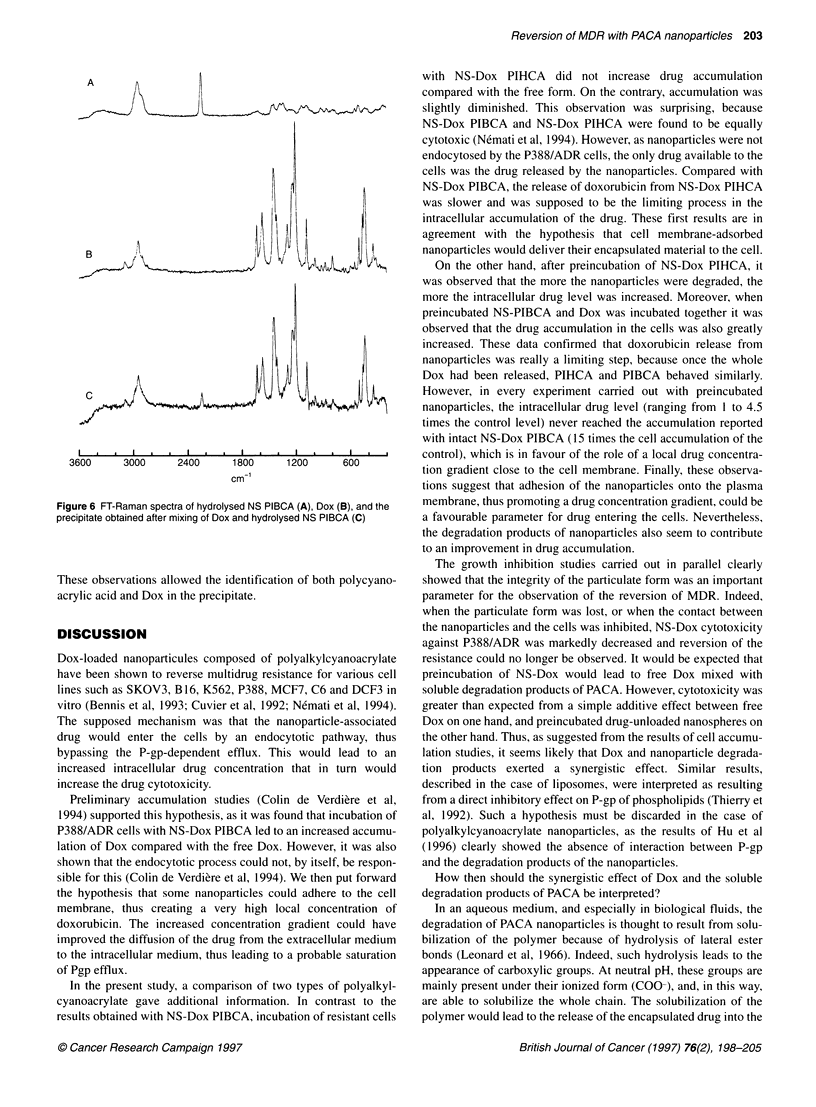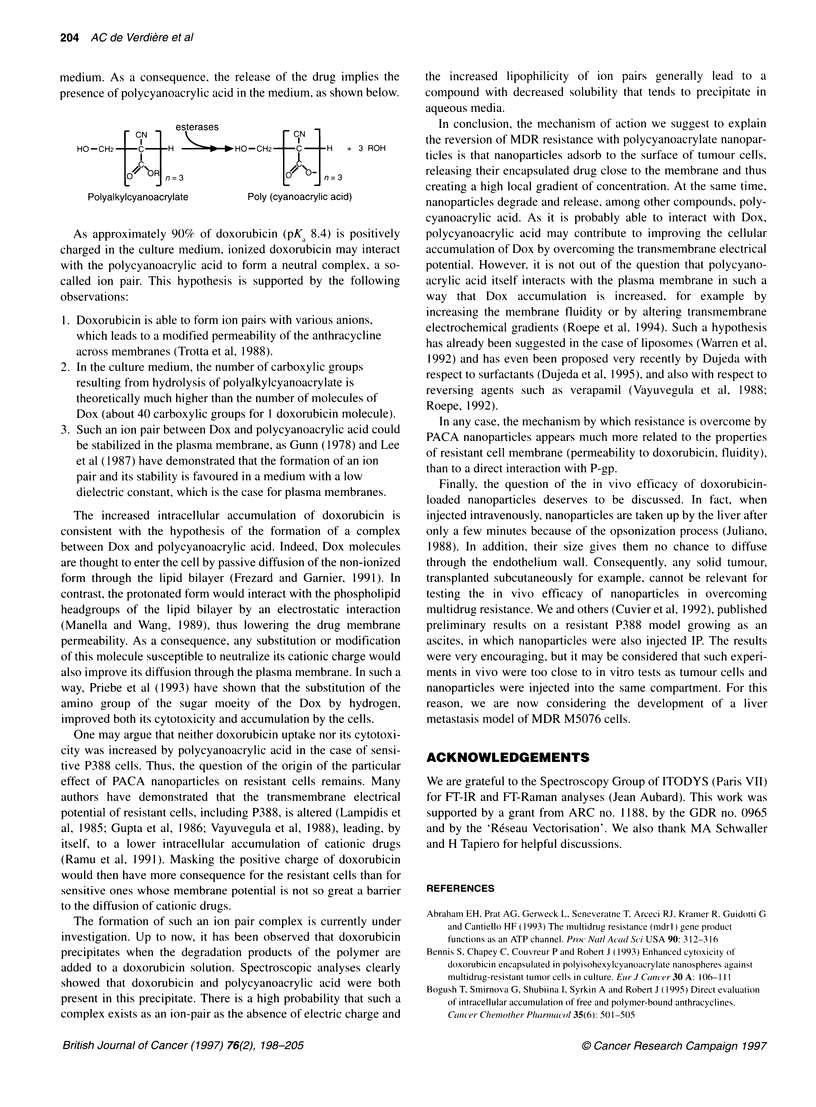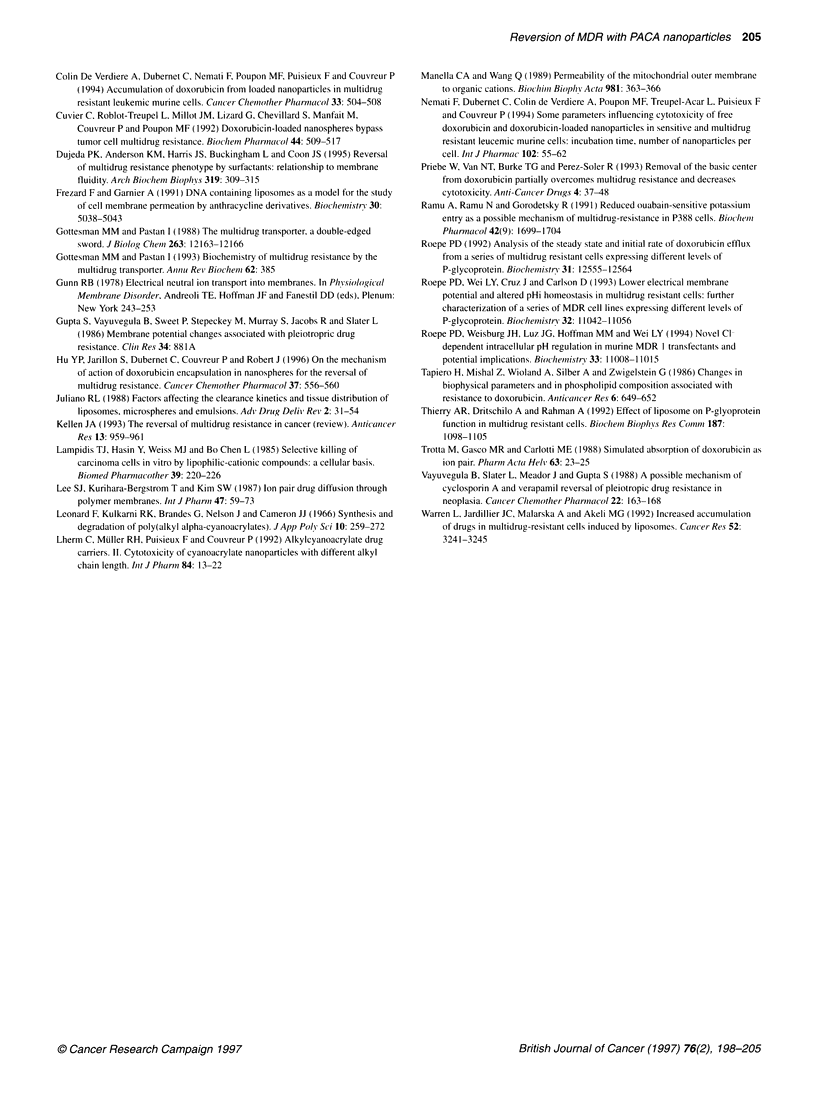# Reversion of multidrug resistance with polyalkylcyanoacrylate nanoparticles: towards a mechanism of action.

**DOI:** 10.1038/bjc.1997.362

**Published:** 1997

**Authors:** A. C. de VerdiÃ¨re, C. Dubernet, F. NÃ©mati, E. Soma, M. Appel, J. FertÃ©, S. Bernard, F. Puisieux, P. Couvreur

**Affiliations:** Centre d'Etudes Pharmaceutiques, URA CNRS 1218, ChÃ¢tenay-Malabry.

## Abstract

Polyalkylcyanoacrylate (PACA) nanoparticles loaded with doxorubicin allowed multidrug resistance to be overcome in vitro. However, increased cytotoxicity is not always correlated with an increased level of intracellular drug. Although we have previously shown that PACA nanoparticles are not endocytosed by tumour cells, we report here that a direct interaction between nanoparticles and cells is a necessary requirement for overcoming resistance. In addition, the results showed that the degradation products of PACA (mainly polycyanoacrylic acid) in the presence of doxorubicin are able to increase both accumulation and cytotoxicity, thus suggesting the formation of a doxorubicin-polycyanoacrylic acid ion pair. It is therefore concluded that resistance is overcome as a result of both the adsorption of nanoparticles to the cell surface and increased doxorubicin diffusion by the accumulation of an ion pair at the plasma membrane.


British Journal of Cancer (1997) 76(2), 198–205
© 1997 Cancer Research Campaign

### Reversion of multidrug resistance with polyalkylcyanoacrylate nanoparticles: towards a mechanism of action

**AC de Verdière[1], C Dubernet[1], F Némati[1], E Soma[1], M Appel[1], J Ferté[2], S Bernard[2], F Puisieux[2], P Couvreur[2]**

[1]Centre d'Etudes Pharmaceutiques, URA CNRS 1218, 5 rue J.B. Clément, 92296 Châtenay-Malabry Cedex; [2]Centre d'Etudes Pharmaceutiques, Biocis URA CNRS 1843, 5 rue J.B. Clément, 92296 Châtenay-Malabry Cedex

**Summary** Polyalkylcyanoacrylate (PACA) nanoparticles loaded with doxorubicin allowed multidrug resistance to be overcome in vitro. However, increased cytotoxicity is not always correlated with an increased level of intracellular drug. Although we have previously shown that PACA nanoparticles are not endocytosed by tumour cells, we report here that a direct interaction between nanoparticles and cells is a necessary requirement for overcoming resistance. In addition, the results showed that the degradation products of PACA (mainly polycyanoacrylic acid) in the presence of doxorubicin are able to increase both accumulation and cytotoxicity, thus suggesting the formation of a doxorubicin–polycyanoacrylic acid ion pair. It is therefore concluded that resistance is overcome as a result of both the adsorption of nanoparticles to the cell surface and increased doxorubicin diffusion by the accumulation of an ion pair at the plasma membrane.

**Keywords:** nanoparticle; polyalkylcyanoacrylate; doxorubicin; multidrug resistance; ion pair; P388

During the past few years, much attention has been given to the mechanisms by which cancer cells become resistant to doxorubicin (Dox). Most of the studies have focused on the P-glycoprotein (P-gp)-dependent mechanism of the (MDR) phenotype. It has been established that P-gp is a membrane glycoprotein of about 170 kDa that is thought to expel drugs from the intracellular to the extracellular medium, via an ATP-dependent mechanism (Gottesman and Pastan, 1993). Until now, this protein has been supposed to be a drug transporter (Gottesman and Pastan, 1993), a flippase (Gottesman and Pastan, 1988) and an ATP channel (Abraham et al, 1993). Moreover, it has recently been established that P-gp expression can alter both intracellular pH and the transmembrane electrical potential, which can affect cationic drug accumulation by resistant cells (Roepe et al, 1993).

Several strategies have been investigated to overcome MDR. Among them, chemical modifications of drugs (Tapiero et al, 1986), co-administration of chemosensitizing compounds, generally acting as P-gp inhibitors (Kellen, 1993), and the use of drug carriers such as microspheres, liposomes or nanoparticles can be mentioned. As far as polymeric nanoparticles are concerned, Cuvier et al (1992) and Nemati et al (1994) have shown that Dox-loaded nanoparticles of polyalkylcyanoacrylate (NS-Dox) are able to bypass multidrug resistance in vitro. Furthermore, preliminary in vivo experiments have given very promising results (Cuvier et al, 1992). In a previous work, we found that the reversion of the resistance of the leukaemic cell line P388/ADR by NS-Dox composed of polyisobutylcyanoacrylate (PIBCA) was related to an increased accumulation of drug by the cells (Colin de Verdière et al, 1994). However, the exact mechanism by which these nanoparticles overcame resistance of cells in culture was not clear. In order to obtain more information, further studies have been carried out with nanoparticles composed of polyisohexylcyanoacrylate (PIHCA), described in this paper. In contrast to PIBCA nanoparticles, with PIHCA nanoparticles no increase in drug accumulation was found. Similar results have been described by Bogush et al (1995). The aim of the present study was to determine how two such very similar polymer nanoparticles overcome the resistance of cells with two completely different effects on the drug accumulation.

*Received 7 May 1996*
*Revised 22 January 1997*
*Accepted 28 January 1997*

*Correspondence to:* C Dubernet

### MATERIALS AND METHODS

### Cell line and culture

P388 (sensitive cells) and P388/ADR (resistant cell line) were kindly supplied by the Institut de Recherche sur le Cancer (IRSC, France). They were grown in suspension, in RPMI-1640 medium (Gibco, France), supplemented with 10% fetal calf serum (Gibco, France), penicillin–streptomycin (Eurobio, France) and 3 nM 2-mercaptoethanol (Sigma, USA).

### Chemicals

Free Dox (Adriblastin) was obtained from Farmitalia (Carlo Erba, Italy). The monomer isohexylcyanoacrylate (IHCA) was kindly supplied by SOPAR (Belgium). The monomer isobutylcyanoacrylate (IBCA) was obtained from Sigma. All other chemicals were obtained commercially and were of analytical grade.

### Preparation of nanoparticles

Nanoparticles were prepared as described previously (Colin de Verdière et al, 1994). Typically, 66.5 mg of monomer (IBCA or IHCA) was dropped under mechanical stirring into 6.5 ml of

**Figure 1** In vitro Dox release from NS-Dox PIBCA (○) and NS-Dox PIHCA (■) as a function of incubation time in cell culture medium. The concentration was 2000 ng ml$^{-1}$

medium containing 5 mg of doxorubicin, 5% glucose, 1% dextran 70 and 0.5% citric acid. After a 24-h (IHCA) or a 6-h (IBCA) polymerization, nanoparticles were obtained and lyophilized. Polymerization times were chosen with respect to the polymerization rate of the monomer, which increases as the alkyl chain length shortens. The final characteristics of the nanoparticles obtained in terms of particle size or average molecular weight were similar for PIHCA and PIBCA nanoparticles. The percentage of doxorubicin associated with the nanoparticles was 95% after resuspension in distilled water. The size of the nanoparticles was determined by quasielastic light scattering in a Nanosizer N4MD (Coultronics, France) and was 186 ± 31 nm for NS-Dox PIBCA and 243 ± 15 nm for NS-Dox PIHCA. Non-loaded nanoparticles (NS-PIBCA and NS-PIHCA) were obtained by the same method, in the absence of doxorubicin in the polymerization medium.

### In vitro release of Dox

The rate of Dox release from NS-Dox PIBCA and NS-Dox PIHCA was measured in vitro. The drug-loaded nanoparticles were incubated in the culture medium at a Dox concentration of 2 μg ml$^{-1}$ at 37°C in a humidified atmosphere containing 5% carbon dioxide. Free Dox was incubated under the same conditions as a control. Samples were taken after various incubation periods and nanoparticles were separated from the released drug by ultrafiltration on a polysulphone membrane (300 000 Da molecular weight cut-off; Millipore, France) at 2000 *g* for 5 min. The filtrate was assayed by HPLC as described in Colin de Verdière et al, 1994.

### Studies of drug accumulation by cells in culture

Cells were seeded at a density of 5 × 10$^5$ cells ml$^{-1}$. After 22 h, Dox, NS-Dox PIBCA or NS-Dox PIHCA were added to the culture medium in order to reach a final concentration of 0.1 μg ml$^{-1}$ for sensitive P388 cells and 2 μg ml$^{-1}$ for resistant P388/ADR cells.

In some experiments, using P388/ADR cells only, we also measured the accumulation of doxorubicin after preincubation of the nanoparticles (with or without loaded drug) in cell culture medium (NS-PIBCA, NS-Dox PIBCA, NS-Dox PIHCA and Dox as control). This preincubation led to the more or less complete bioerosion of the nanoparticles, depending on the duration for which it was performed. Preincubation was carried out at a Dox concentration of 2 μg ml$^{-1}$, corresponding to a polymer concentration of 26 μg ml$^{-1}$ for different periods of time (1 h for NS-Dox PIBCA and NS-PIBCA and 3 h or overnight for NS-Dox PIHCA). Thereafter, the contents of wells, which had been previously seeded with cells, were centrifuged and the supernatants were discarded. The cell pellets were then resuspended in medium containing the preincubated nanoparticles. After varying periods of incubation with the drug, cell-associated Dox was determined as described by Colin de Verdière et al (1994).

### Inhibition of cell growth

The following were assessed for their ability to inhibit cell growth: free Dox, Dox-loaded nanoparticles made of PIHCA (NS-Dox PIHCA) or PIBCA (NS-Dox PIBCA), unloaded nanoparticles (NS) and a mixture of free Dox and unloaded nanoparticles (NS+Dox). Cells (P388 and P388/ADR) were incubated for 48 h at 5 × 10$^4$ cells ml$^{-1}$ with various concentrations of the drug. The resulting cell number was determined in a model ZM Coulter counter (Coultronics, France). Some experiments were carried out in special wells (Costar, France) consisting of two compartments separated by a porous membrane (polycarbonate, pore size 0.1 μm). Cells were seeded in the lower compartment and the drug samples were added to the upper part, leading to the absence of direct contact between the cells and the nanoparticles. Lastly, in some experiments, we also measured the inhibition of cell growth after preincubation of the nanoparticles (drug-loaded or not) in the cell culture medium as described above.

All the experiments of inhibition of cell growth were carried out on both sensitive and resistant P388 cells. For each one of the figures included in the results section, the error bars correspond to the intraexperimental variations (mean of three different wells). Each experiment was repeated three times with similar results.

### Spectroscopic analysis of doxorubicin

The existence of a complex between Dox and poly(cyanoacrylic acid) was sought using FT-IR and FT-Raman spectroscopy. Poly(cyanoacrylic acid) was obtained as follows: NS-PIBCA were washed three times with distilled water and ultracentrifuged. Then, 0.5 ml of 0.2 N sodium hydroxide was added to 1 ml of a suspension containing 10 mg ml$^{-1}$ NS-PIBCA. Forty-eight hours later, the pH was adjusted to 7.4 with 1 N hydrochloric acid. An aliquot (145 μl) of an aqueous Dox solution (5 mg ml$^{-1}$) was then added to the hydrolysed polymer: a red precipitate appeared instantanously. After centrifugation at 15 400 *g* (Sigma 2K15), the pellet was resuspended in 100 μl of distilled water and lyophilized.

The dried solid was analysed by FT-IR (Nicolet SX60) and FT-Raman (Perkin Elmer System 2000, λ = 1060 nm, YAG laser).

### RESULTS

### In vitro release studies

As shown in Figure 1, Dox release from nanoparticles in culture medium was much faster in the case of PIBCA nanoparticles than in the case of PIHCA ones. Drug release from PIBCA started immediately and was complete within 1 h. In the case of PIHCA, the release displayed a biphasic character: no significant release occurred within the first hour, and complete release was only observed after 400 min.

© Cancer Research Campaign 1997 

**Figure 2** Amount of Dox associated with P388/ADR cells as a function of time of incubation. The drug was added at a concentration of 2 μg ml$^{-1}$ either as free doxorubicin (Dox) or associated with nanoparticles: NS-Dox PIBCA (**A**); NS-Dox PIHCA (**B**); preincubated NS-Dox PIBCA (NS-Dox PIBCA 1 h) (**C**); a mixture of preincubated NS-PIBCA and free doxorubicin (NS-PIBCA deg + Dox) (**D**); and finally preincubated NS-Dox PIHCA (NS-Dox PIHCA 3 h and NS-Dox PIHCA overnight) (**E**)

### Drug accumulation in cells in culture

### *Cell accumulation experiments with NS-Dox PIBCA and NS-Dox PIHCA*

As described in Materials and methods, cells were incubated with Dox, NS-Dox PIBCA and NS-Dox PIHCA. In the case of sensitive P388 cells, incubated in the presence of 0.1 μg ml$^{-1}$ doxorubicin, the drug accumulation level was the same whatever the drug formulation (free Dox or NS-Dox). Similar results have already been published (Colin de Verdière et al, 1994). When P388/ADR cells were incubated with Dox at a concentration of 2 μg ml$^{-1}$, the accumulation quickly reached a steady state, corresponding to 20 ng doxorubicin per million cells (Figure 2A). When incubated with PIBCA nanoparticles, doxorubicin accumulation was increased to 300 ng per million cells, which represents about 15 times the level obtained after a 6-h incubation with free Dox.

Surprisingly, when PIHCA nanoparticles were used, drug accumulation was very low (Figure 2B) and not significantly different from that observed after incubation with free Dox. This result is inconsistent with the data reported in previous cytotoxicity experiments, showing that NS-Dox PIBCA and NS-Dox PIHCA were equally cytotoxic (Némati et al, 1994). This comparison suggests that cytotoxic effects of nanoparticles are not a direct consequence of drug accumulation within cells.

 © Cancer Research Campaign 1997

**Figure 3** Cytotoxicity against P388 (**A**) and P388/ADR (**B**) of PIHCA nanoparticles in a two-compartment well (**a**) without the presence of the separating membrane and (**b**) in the presence of the separating membrane

### *Cell accumulation experiments with preincubated nanoparticles (NS-Dox PIBCA and NS-Dox PIHCA)*

When NS-Dox PIBCA were preincubated for 1 h in the culture medium before incubation with the cells, the accumulation of Dox did not differ markedly from the accumulation obtained with non-degraded NS-Dox PIBCA (Figure 2C). After a 6 h incubation, no equilibrium was reached and the amount of drug associated with the cells was 220 ng per million cells (i.e. 3.6 times the level obtained after an incubation with free Dox).

In cell accumulation experiments with the degradation products of NS-Dox PIHCA, the amount of drug associated with the cells depended on the time of preincubation of the nanoparticles (Figure 2E): after a period of incubation of 3 h, which corresponds to 50% release of the drug in the cell culture medium (see Figure 1), cell accumulation of doxorubicin was not significantly increased compared with intact NS-Dox PIHCA.

In contrast, after complete release of the drug from the nanoparticles, achieved by an overnight preincubation of NS-Dox PIHCA, the accumulation of the drug by the cells was significantly increased and reached a value of 130 ng per million of cells after a 6-h incubation time. This represented approximately 2.6 times the Dox cell accumulation value of the control (Dox) and 3.2 times the accumulation level of intact NS-Dox PIHCA.

### *Cell accumulation experiment with a mixture of preincubated NS-PIBCA and Dox*

When P388/ADR cells were incubated with a mixture of the degradation products of NS-PIBCA and free Dox, a considerable increase in the accumulation of drug by the cells was observed, as compared to the free Dox (Figure 2D). After a 6 h incubation, accumulation of the drug reached approximatively 450 ng per million cells, which is close to 4.5 times the value of the control (100 ng per million cells for Dox).

### Inhibition of cell growth

### *Two-compartment well experiments*

Cells were seeded in the lower compartment of the wells and could be separated from the drug compartment by a mobile porous membrane. When the experiment was carried out with sensitive

**Figure 4** Cytotoxicity against P388/ADR of PIHCA and PIBCA nanoparticles (non-loaded and doxorubicin-loaded) as a function of the time of preincubation of the nanospheres before their addition to the cells. Dox concentration was 200 ng ml$^{-1}$

**Figure 5** Cytotoxicity against P388 (**A**) P388/ADR (**B**) and of free Dox, NS-Dox PIBCA, NS-PIBCA and completely bioeroded nanoparticles (NS-preincub, NS-Dox preincub and a mixture of free Dox and NS-preincub)

P388 cells (Figure 3A), it was observed that Dox and NS-Dox (NS-Dox PIHCA in the figure) were equally cytotoxic in the absence (Figure 3Aa) or in the presence (Figure 3Ab) of the membrane. The only difference was due to a partial adsorption of doxorubicin onto the membrane, leading to higher $IC_{50}$ in the presence of the separating membrane (Figure 3Ab as compared with Figure 3Aa). It was also noted that unloaded nanospheres (NS-PIHCA) appeared to be less cytotoxic towards P388 cells when direct contact between nanoparticles and cells was inhibited by the porous membrane. This observation has already been discussed elsewhere (Lherm et al, 1992) and was attributed to a membrane cytotoxic effect of the degradation products of the polymer released and concentrated close to the cell surface when nanoparticles were adsorbed onto the cells.

As far as P388/ADR cells were concerned, when they were not in direct contact with the drug (Figure 3Bb), NS-Dox PIHCA appeared to be as cytotoxic as the free drug, and non-loaded nanoparticles appeared non-cytotoxic at the doses investigated. Thus, the presence of the separating membrane led to a sharp decrease in the cytotoxicity of the nanoparticles (as compared with studies performed in the absence of the membrane; Figure 3Ba).

### *Experiments performed with preincubated nanoparticles*

Nanoparticles were incubated in the culture medium before addition to the cells. Figure 4 shows that, in the case of P388/ADR cells, the particle cytotoxicity was decreased when NS-Dox was preincubated in the culture medium. Cytotoxicity was completely abolished after a 2-h incubation for PIBCA and after a 6-h incubation for PIHCA. These time periods correspond to the complete bioerosion of the particles, PIBCA being degraded much faster than PIHCA (see Figure 1).

A final set of experiments was carried out with nanoparticles preincubated for a time sufficient to allow complete degradation of nanoparticles. In the case of P388 sensitive cells, there was no difference in cytotoxicity between Dox, NS-Dox or preincubated NS-Dox (Figure 5A), whereas preincubated unloaded nanospheres were less cytotoxic than original non-loaded nanospheres. In the case of P388/ADR cells, the results were more complex. The totally bioeroded NS-Dox particles (NS-Dox preincub) were still more cytotoxic than the free drug (the $IC_{50}$ being 500 ng ml$^{-1}$ and 1500 ng ml$^{-1}$ respectively) although this cytotoxic effect was lower than in the presence of intact particles ($IC_{50}$ around 70 ng ml$^{-1}$) (Figure 5B). It was even shown that a mixture of Dox with the preincubated polymer was more cytotoxic than would have been expected from the simple addition of the cytotoxic effects of the free drug and of the preincubated polymer (Figure 5B). Indeed, at a Dox concentration of 500 ng ml$^{-1}$, the mixture NS preincub + Dox resulted in 50% cytotoxicity, whereas NS preincub alone and Dox alone led to 80% and 85% of the control respectively.

### Spectroscopic analyses

As shown in Figure 6, the Fourier transform (FT) Raman spectrum of the red precipitate obtained when hydrolysed PIBCA was added to a Dox solution, appeared to be the superposition of those of Dox and of the hydrolysed polymer. Of particular interest are the bands observed from 1800 cm$^{-1}$ to 200 cm$^{-1}$, mainly attributed to doxorubicin, and the band at 2250 cm$^{-1}$ corresponding to CN groups, a specific function of polycyanoacrylate. Fourier transform infrared spectroscopy gave similar information, although the spectra were less clear because of the lower intensity of the band at 2250 cm$^{-1}$.

© Cancer Research Campaign 1997

**Figure 6** FT-Raman spectra of hydrolysed NS PIBCA (**A**), Dox (**B**), and the precipitate obtained after mixing of Dox and hydrolysed NS PIBCA (**C**)

These observations allowed the identification of both polycyanoacrylic acid and Dox in the precipitate.

### DISCUSSION

Dox-loaded nanoparticules composed of polyalkylcyanoacrylate have been shown to reverse multidrug resistance for various cell lines such as SKOV3, B16, K562, P388, MCF7, C6 and DCF3 in vitro (Bennis et al, 1993; Cuvier et al, 1992; Némati et al, 1994). The supposed mechanism was that the nanoparticle-associated drug would enter the cells by an endocytotic pathway, thus bypassing the P-gp-dependent efflux. This would lead to an increased intracellular drug concentration that in turn would increase the drug cytotoxicity.

Preliminary accumulation studies (Colin de Verdière et al, 1994) supported this hypothesis, as it was found that incubation of P388/ADR cells with NS-Dox PIBCA led to an increased accumulation of Dox compared with the free Dox. However, it was also shown that the endocytotic process could not, by itself, be responsible for this (Colin de Verdière et al, 1994). We then put forward the hypothesis that some nanoparticles could adhere to the cell membrane, thus creating a very high local concentration of doxorubicin. The increased concentration gradient could have improved the diffusion of the drug from the extracellular medium to the intracellular medium, thus leading to a probable saturation of Pgp efflux.

In the present study, a comparison of two types of polyalkylcyanoacrylate gave additional information. In contrast to the results obtained with NS-Dox PIBCA, incubation of resistant cells with NS-Dox PIHCA did not increase drug accumulation compared with the free form. On the contrary, accumulation was slightly diminished. This observation was surprising, because NS-Dox PIBCA and NS-Dox PIHCA were found to be equally cytotoxic (Némati et al, 1994). However, as nanoparticles were not endocytosed by the P388/ADR cells, the only drug available to the cells was the drug released by the nanoparticles. Compared with NS-Dox PIBCA, the release of doxorubicin from NS-Dox PIHCA was slower and was supposed to be the limiting process in the intracellular accumulation of the drug. These first results are in agreement with the hypothesis that cell membrane-adsorbed nanoparticles would deliver their encapsulated material to the cell.

On the other hand, after preincubation of NS-Dox PIHCA, it was observed that the more the nanoparticles were degraded, the more the intracellular drug level was increased. Moreover, when preincubated NS-PIBCA and Dox was incubated together it was observed that the drug accumulation in the cells was also greatly increased. These data confirmed that doxorubicin release from nanoparticles was really a limiting step, because once the whole Dox had been released, PIHCA and PIBCA behaved similarly. However, in every experiment carried out with preincubated nanoparticles, the intracellular drug level (ranging from 1 to 4.5 times the control level) never reached the accumulation reported with intact NS-Dox PIBCA (15 times the cell accumulation of the control), which is in favour of the role of a local drug concentration gradient close to the cell membrane. Finally, these observations suggest that adhesion of the nanoparticles onto the plasma membrane, thus promoting a drug concentration gradient, could be a favourable parameter for drug entering the cells. Nevertheless, the degradation products of nanoparticles also seem to contribute to an improvement in drug accumulation.

The growth inhibition studies carried out in parallel clearly showed that the integrity of the particulate form was an important parameter for the observation of the reversion of MDR. Indeed, when the particulate form was lost, or when the contact between the nanoparticles and the cells was inhibited, NS-Dox cytotoxicity against P388/ADR was markedly decreased and reversion of the resistance could no longer be observed. It would be expected that preincubation of NS-Dox would lead to free Dox mixed with soluble degradation products of PACA. However, cytotoxicity was greater than expected from a simple additive effect between free Dox on one hand, and preincubated drug-unloaded nanospheres on the other hand. Thus, as suggested from the results of cell accumulation studies, it seems likely that Dox and nanoparticle degradation products exerted a synergistic effect. Similar results, described in the case of liposomes, were interpreted as resulting from a direct inhibitory effect on P-gp of phospholipids (Thierry et al, 1992). Such a hypothesis must be discarded in the case of polyalkylcyanoacrylate nanoparticles, as the results of Hu et al (1996) clearly showed the absence of interaction between P-gp and the degradation products of the nanoparticles.

How then should the synergistic effect of Dox and the soluble degradation products of PACA be interpreted?

In an aqueous medium, and especially in biological fluids, the degradation of PACA nanoparticles is thought to result from solubilization of the polymer because of hydrolysis of lateral ester bonds (Leonard et al, 1966). Indeed, such hydrolysis leads to the appearance of carboxylic groups. At neutral pH, these groups are mainly present under their ionized form ($COO^-$), and, in this way, are able to solubilize the whole chain. The solubilization of the polymer would lead to the release of the encapsulated drug into the medium. As a consequence, the release of the drug implies the presence of polycyanoacrylic acid in the medium, as shown below.

As approximately 90% of doxorubicin (p$K_a$ 8.4) is positively charged in the culture medium, ionized doxorubicin may interact with the polycyanoacrylic acid to form a neutral complex, a so-called ion pair. This hypothesis is supported by the following observations:

1. Doxorubicin is able to form ion pairs with various anions, which leads to a modified permeability of the anthracycline across membranes (Trotta et al, 1988).
2. In the culture medium, the number of carboxylic groups resulting from hydrolysis of polyalkylcyanoacrylate is theoretically much higher than the number of molecules of Dox (about 40 carboxylic groups for 1 doxorubicin molecule).
3. Such an ion pair between Dox and polycyanoacrylic acid could be stabilized in the plasma membrane, as Gunn (1978) and Lee et al (1987) have demonstrated that the formation of an ion pair and its stability is favoured in a medium with a low dielectric constant, which is the case for plasma membranes.

The increased intracellular accumulation of doxorubicin is consistent with the hypothesis of the formation of a complex between Dox and polycyanoacrylic acid. Indeed, Dox molecules are thought to enter the cell by passive diffusion of the non-ionized form through the lipid bilayer (Frezard and Garnier, 1991). In contrast, the protonated form would interact with the phospholipid headgroups of the lipid bilayer by an electrostatic interaction (Manella and Wang, 1989), thus lowering the drug membrane permeability. As a consequence, any substitution or modification of this molecule susceptible to neutralize its cationic charge would also improve its diffusion through the plasma membrane. In such a way, Priebe et al (1993) have shown that the substitution of the amino group of the sugar moeity of the Dox by hydrogen, improved both its cytotoxicity and accumulation by the cells.

One may argue that neither doxorubicin uptake nor its cytotoxicity was increased by polycyanoacrylic acid in the case of sensitive P388 cells. Thus, the question of the origin of the particular effect of PACA nanoparticles on resistant cells remains. Many authors have demonstrated that the transmembrane electrical potential of resistant cells, including P388, is altered (Lampidis et al, 1985; Gupta et al, 1986; Vayuvegula et al, 1988), leading, by itself, to a lower intracellular accumulation of cationic drugs (Ramu et al, 1991). Masking the positive charge of doxorubicin would then have more consequence for the resistant cells than for sensitive ones whose membrane potential is not so great a barrier to the diffusion of cationic drugs.

The formation of such an ion pair complex is currently under investigation. Up to now, it has been observed that doxorubicin precipitates when the degradation products of the polymer are added to a doxorubicin solution. Spectroscopic analyses clearly showed that doxorubicin and polycyanoacrylic acid were both present in this precipitate. There is a high probability that such a complex exists as an ion-pair as the absence of electric charge and the increased lipophilicity of ion pairs generally lead to a compound with decreased solubility that tends to precipitate in aqueous media.

In conclusion, the mechanism of action we suggest to explain the reversion of MDR resistance with polycyanoacrylate nanoparticles is that nanoparticles adsorb to the surface of tumour cells, releasing their encapsulated drug close to the membrane and thus creating a high local gradient of concentration. At the same time, nanoparticles degrade and release, among other compounds, polycyanoacrylic acid. As it is probably able to interact with Dox, polycyanoacrylic acid may contribute to improving the cellular accumulation of Dox by overcoming the transmembrane electrical potential. However, it is not out of the question that polycyanoacrylic acid itself interacts with the plasma membrane in such a way that Dox accumulation is increased, for example by increasing the membrane fluidity or by altering transmembrane electrochemical gradients (Roepe et al, 1994). Such a hypothesis has already been suggested in the case of liposomes (Warren et al, 1992) and has even been proposed very recently by Dujeda with respect to surfactants (Dujeda et al, 1995), and also with respect to reversing agents such as verapamil (Vayuvegula et al, 1988; Roepe, 1992).

In any case, the mechanism by which resistance is overcome by PACA nanoparticles appears much more related to the properties of resistant cell membrane (permeability to doxorubicin, fluidity), than to a direct interaction with P-gp.

Finally, the question of the in vivo efficacy of doxorubicin-loaded nanoparticles deserves to be discussed. In fact, when injected intravenously, nanoparticles are taken up by the liver after only a few minutes because of the opsonization process (Juliano, 1988). In addition, their size gives them no chance to diffuse through the endothelium wall. Consequently, any solid tumour, transplanted subcutaneously for example, cannot be relevant for testing the in vivo efficacy of nanoparticles in overcoming multidrug resistance. We and others (Cuvier et al, 1992), published preliminary results on a resistant P388 model growing as an ascites, in which nanoparticles were also injected IP. The results were very encouraging, but it may be considered that such experiments in vivo were too close to in vitro tests as tumour cells and nanoparticles were injected into the same compartment. For this reason, we are now considering the development of a liver metastasis model of MDR M5076 cells.

### ACKNOWLEDGEMENTS

We are grateful to the Spectroscopy Group of ITODYS (Paris VII) for FT-IR and FT-Raman analyses (Jean Aubard). This work was supported by a grant from ARC no. 1188, by the GDR no. 0965 and by the 'Réseau Vectorisation'. We also thank MA Schwaller and H Tapiero for helpful discussions.

### REFERENCES

Abraham EH, Prat AG, Gerweck L, Seneveratne T, Arceci RJ, Kramer R, Guidotti G and Cantiello HF (1993) The multidrug resistance (mdr1) gene product functions as an ATP channel. *Proc Natl Acad Sci* USA **90**: 312–316

Bennis S, Chapey C, Couvreur P and Robert J (1993) Enhanced cytoxicity of doxorubicin encapsulated in polyisohexylcyanoacrylate nanospheres against multidrug-resistant tumor cells in culture. *Eur J Cancer* **30 A**: 106–111

Bogush T, Smirnova G, Shubiina I, Syrkin A and Robert J (1995) Direct evaluation of intracellular accumulation of free and polymer-bound anthracyclines. *Cancer Chemother Pharmacol* **35**(6): 501–505

© Cancer Research Campaign 1997

Colin De Verdiere A, Dubernet C, Nemati F, Poupon MF, Puisieux F and Couvreur P (1994) Accumulation of doxorubicin from loaded nanoparticles in multidrug resistant leukemic murine cells. *Cancer Chemother Pharmacol* **33**: 504–508

Cuvier C, Roblot-Treupel L, Millot JM, Lizard G, Chevillard S, Manfait M, Couvreur P and Poupon MF (1992) Doxorubicin-loaded nanospheres bypass tumor cell multidrug resistance. *Biochem Pharmacol* **44**: 509–517

Dujeda PK, Anderson KM, Harris JS, Buckingham L and Coon JS (1995) Reversal of multidrug resistance phenotype by surfactants: relationship to membrane fluidity. *Arch Biochem Biophys* **319**: 309–315

Frezard F and Garnier A (1991) DNA containing liposomes as a model for the study of cell membrane permeation by anthracycline derivatives. *Biochemistry* **30**: 5038–5043

Gottesman MM and Pastan I (1988) The multidrug transporter, a double-edged sword. *J Biolog Chem* **263**: 12163–12166

Gottesman MM and Pastan I (1993) Biochemistry of multidrug resistance by the multidrug transporter. *Annu Rev Biochem* **62**: 385

Gunn RB (1978) Electrical neutral ion transport into membranes. In *Physiological Membrane Disorder*, Andreoli TE, Hoffman JF and Fanestil DD (eds), Plenum: New York 243–253

Gupta S, Vayuvegula B, Sweet P, Stepeckey M, Murray S, Jacobs R and Slater L (1986) Membrane potential changes associated with pleiotropric drug resistance. *Clin Res* **34**: 881A

Hu YP, Jarillon S, Dubernet C, Couvreur P and Robert J (1996) On the mechanism of action of doxorubicin encapsulation in nanospheres for the reversal of multidrug resistance. *Cancer Chemother Pharmacol* **37**: 556–560

Juliano RL (1988) Factors affecting the clearance kinetics and tissue distribution of liposomes, microspheres and emulsions. *Adv Drug Deliv Rev* **2**: 31–54

Kellen JA (1993) The reversal of multidrug resistance in cancer (review). *Anticancer Res* **13**: 959–961

Lampidis TJ, Hasin Y, Weiss MJ and Bo Chen L (1985) Selective killing of carcinoma cells in vitro by lipophilic-cationic compounds: a cellular basis. *Biomed Pharmacother* **39**: 220–226

Lee SJ, Kurihara-Bergstrom T and Kim SW (1987) Ion pair drug diffusion through polymer membranes. *Int J Pharm* **47**: 59–73

Leonard F, Kulkarni RK, Brandes G, Nelson J and Cameron JJ (1966) Synthesis and degradation of poly(alkyl alpha-cyanoacrylates). *J App Poly Sci* **10**: 259–272

Lherm C, Müller RH, Puisieux F and Couvreur P (1992) Alkylcyanoacrylate drug carriers. II. Cytotoxicity of cyanoacrylate nanoparticles with different alkyl chain length. *Int J Pharm* **84**: 13–22

Manella CA and Wang Q (1989) Permeability of the mitochondrial outer membrane to organic cations. *Biochim Biophy Acta* **981**: 363–366

Nemati F, Dubernet C, Colin de Verdiere A, Poupon MF, Treupel-Acar L, Puisieux F and Couvreur P (1994) Some parameters influencing cytotoxicity of free doxorubicin and doxorubicin-loaded nanoparticles in sensitive and multidrug resistant leucemic murine cells: incubation time, number of nanoparticles per cell. *Int J Pharmac* **102**: 55–62

Priebe W, Van NT, Burke TG and Perez-Soler R (1993) Removal of the basic center from doxorubicin partially overcomes multidrug resistance and decreases cytotoxicity. *Anti-Cancer Drugs* **4**: 37–48

Ramu A, Ramu N and Gorodetsky R (1991) Reduced ouabain-sensitive potassium entry as a possible mechanism of multidrug-resistance in P388 cells. *Biochem Pharmacol* **42**(9): 1699–1704

Roepe PD (1992) Analysis of the steady state and initial rate of doxorubicin efflux from a series of multidrug resistant cells expressing different levels of P-glycoprotein. *Biochemistry* **31**: 12555–12564

Roepe PD, Wei LY, Cruz J and Carlson D (1993) Lower electrical membrane potential and altered pHi homeostasis in multidrug resistant cells: further characterization of a series of MDR cell lines expressing different levels of P-glycoprotein. *Biochemistry* **32**: 11042–11056

Roepe PD, Weisburg JH, Luz JG, Hoffman MM and Wei LY (1994) Novel $Cl^-$ dependent intracellular pH regulation in murine MDR 1 transfectants and potential implications. *Biochemistry* **33**: 11008–11015

Tapiero H, Mishal Z, Wioland A, Silber A and Zwigelstein G (1986) Changes in biophysical parameters and in phospholipid composition associated with resistance to doxorubicin. *Anticancer Res* **6**: 649–652

Thierry AR, Dritschilo A and Rahman A (1992) Effect of liposome on P-glyoprotein function in multidrug resistant cells. *Biochem Biophys Res Comm* **187**: 1098–1105

Trotta M, Gasco MR and Carlotti ME (1988) Simulated absorption of doxorubicin as ion pair. *Pharm Acta Helv* **63**: 23–25

Vayuvegula B, Slater L, Meador J and Gupta S (1988) A possible mechanism of cyclosporin A and verapamil reversal of pleiotropic drug resistance in neoplasia. *Cancer Chemother Pharmacol* **22**: 163–168

Warren L, Jardillier JC, Malarska A and Akeli MG (1992) Increased accumulation of drugs in multidrug-resistant cells induced by liposomes. *Cancer Res* **52**: 3241–3245

© Cancer Research Campaign 1997